# Lead Concentrations in Relation to Multiple Biomarkers of Cardiovascular Disease: The Normative Aging Study

**DOI:** 10.1289/ehp.1103467

**Published:** 2011-12-05

**Authors:** Junenette L. Peters, Laura D. Kubzansky, Ai Ikeda, Shona C. Fang, David Sparrow, Marc G. Weisskopf, Robert O. Wright, Pantel Vokonas, Howard Hu, Joel Schwartz

**Affiliations:** 1 Department of Environment Health, Harvard School of Public Health, Boston, Massachusetts, USA; 2 Department of Environment Health, Boston University of Public Health, Boston, Massachusetts, USA; 3 Department of Society, Human Development and Health, Harvard School of Public Health, Boston, Massachusetts, USA; 4 Veterans Affairs Boston Healthcare System, and Boston University Schools of Medicine and Public Health, Boston, Massachusetts, USA; 5 Channing Laboratory, Department of Medicine, Brigham and Women’s Hospital, Harvard Medical School, Boston, Massachusetts, USA; 6 Department of Environmental Health Sciences, University of Michigan School of Public Health, Ann Arbor, Michigan, USA

**Keywords:** aging, biomarkers, cardiovascular, cholesterol, inflammation, lead, metals

## Abstract

Background: Lead exposure has been associated with cardiovascular disease (CVD) in animal and human studies. However, the mechanisms of action have not been fully elucidated. We therefore examined the relationship between lead and multiple biomarkers of CVD.

Methods: Participants were older men from the Normative Aging Study without preexisting coronary heart disease, diabetes, or active infection at baseline (*n* = 426). Serum biomarkers included lipid profile [total cholesterol, high-density lipoprotein (HDL), low-density lipoprotein (LDL), and triglycerides] and inflammatory markers [C-reactive protein, intercellular adhesion molecule-1, interleukin-6, and tumor necrosis factor receptor-2 (TNF-R2)]. We measured lead in blood and in bone by K-shell X-ray fluorescence. In this sample, 194 men (44.3%) had two or more repeated measures, resulting in 636 observations for analysis. We conducted analyses using mixed effects models with random subject intercepts.

Results: Lead levels were associated with several CVD biomarkers, including levels of TNF-R2 and lipid markers. Specifically, in multivariable models, a 50% increase in blood lead level was associated with 26% increased odds of high TNF-R2 levels (> 5.52 ng/mL; odds ratio = 1.26; 95% confidence interval: 1.09, 1.45). There were positive associations of blood lead level with total cholesterol and HDL levels, and these associations were more evident when modeled as continuous outcomes than when categorized using clinically relevant cut points. In addition, longitudinal analyses indicated a significant increase in TNF-R2 levels over time to be associated with high blood lead level at the preceding visit.

Conclusions: Blood lead level may be related with CVD in healthy older men through its association with TNF-R2 levels. In addition, the magnitude of the association of blood lead level with TNF-R2 level increased with age in the study population.

There is increasing recognition of the role of environmental pollutants in relation to cardiovascular disease (CVD) (Bhatnagar 2006). Among the environmental factors, lead exposure has been found in human studies to be associated with hypertension, peripheral arterial disease, and circulatory and cardiovascular mortality, and in animal studies, to promote hypertension and accelerate CVD (Bhatnagar 2006; Navas-Acien et al. 2007; Weisskopf et al. 2009). In *in vitro* and *in vivo* studies, lead has been shown to decrease the bioavailability of nitric oxide partially by increasing the production of reactive oxygen species (ROS). It is postulated that the related increase in ROS and changes in cytokine production may promote atherogenesis, although this has not been fully explored (Bhatnagar 2006), particularly in epidemiologic studies.

Atherogenesis is related to several biological processes, among them lipid metabolism and inflammation. Although interrelated in their effects on atherogenesis, these processes are typically considered separately in studies seeking to identify factors that may increase risk of dysfunction in these domains. Experimental studies and human studies on lead poisoning or occupationally exposed workers have produced unexpected findings regarding the effect of lead on lipid metabolism. For example, Cocco et al. (1991) found that lead-exposed patients had decreased cholesterol and low-density lipoprotein (LDL) and increased high-density lipoprotein (HDL) levels. Conversely, Ademuyiwa et al. (2005) reported a significant positive correlation between blood lead and total cholesterol and LDL levels. Among studies of inflammation, *in vitro* studies and a study of occupationally exposed workers found a relationship between lead and tumor necrosis factor-α (TNF-α), but not between lead and interleukin-6 (IL-6) (Valentino et al. 2007). TNF-α is involved in chronic cell-mediated inflammation (Valentino et al. 2007) and is associated with endothelial dysfunction, ROS, and lipid metabolism. Findings for high-sensitivity C-reactive protein (hs-CRP) have been mixed. One occupational study found a significant relation between lead exposure and CRP (Khan et al. 2008), but a population-based study did not find a consistent association (Songdej et al. 2010). We know of no study that has directly investigated the role of lead on endothelial dysfunction [i.e., adhesion molecules such as intercellular adhesion molecule-1 (ICAM-1)].

We examined the relationship between lead levels and a comprehensive suite of cardiovascular risk biomarkers, including lipid profile and inflammatory markers, in a population of older men participating in the Normative Aging Study (NAS). We also investigated the relation over time. We tested the hypothesis that higher levels of blood or bone lead would be associated with higher total cholesterol, LDL, and triglycerides levels, with lower HDL levels, and with higher hs-CRP, ICAM-1, IL-6, and TNF receptor 2 (TNF-R2) levels at specific time-points and across time (i.e., associated with higher rates of change in biomarker levels).

## Methods

*Study population.* NAS is a longitudinal study of aging that was established in 1963 by the Veterans Administration [now the Department of Veterans Affairs (VA)]. The subgroup of participants used in the present analysis has been described elsewhere (Cheng et al. 2001; Hu et al. 1996). Briefly, NAS is a closed cohort of 2,280 male volunteers from the Greater Boston area who were screened at entry and enrolled if they were free of any known chronic medical conditions. Participants have been reevaluated every 3–5 years using questionnaires and detailed on-site physical examinations.

**Figure 1:**
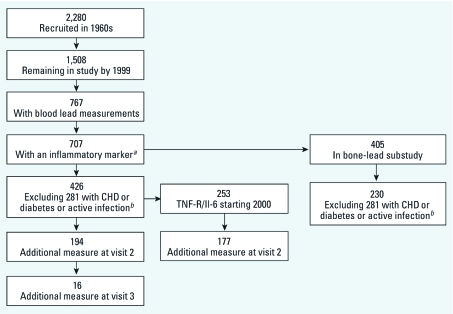
Flow chart of participants. CHD, coronary heart disease. ***^a^***Total cholesterol, LDL, HDL, triglycerides, hs-CRP, ICAM-1. ***^b^***hs-CRP > 10 mg/L.

By 1999, when measurements of inflammatory markers began, 668 original participants had died, and 104 others were no longer being followed primarily because they had moved out of the region after retirement. Further, some participants were lost during recruitment into the lead substudy, leaving 767 participants with blood lead measurements taken during the 1999–2008 study period. Of the 767 men, we had serum measurements of lipids or inflammatory markers on 707 during this period (see Figure 1 for participant flow chart).

At baseline (time of their first inflammatory measure), 198 men with coronary heart disease (i.e., angina pectoris or history of myocardial infarction), 57 men with diabetes mellitus (fasting glucose ≥ 126 mg/dL or medication use), and 26 men with hs-CRP > 10 mg/L (indicating active infection) were excluded, resulting in a study population of 426 men. Exclusion by disease status at baseline is based on the assumption that lead may affect the selected biomarkers in part through its effects on these diseases, which would make them causal intermediates. Among these 426 men, 16 (2.52%) had two additional visits, 178 (41.8%) had one additional visit, and 232 (54.5%) had only one visit over the course of the study period, yielding a total of 636 observations with biomarker and blood lead data available for analysis. However, the number of observations varied among biomarkers because of differences in the starting year of the measurements (Figure 1).

Beginning in 1991, participants were invited to undergo bone lead measurements at the Ambulatory Clinical Research Center of the Brigham and Women’s Hospital (Harvard School of Public Health, Boston, Massachusetts), and these measurements continued until 2002. Not all participants agreed to these extra visits. Of the 707 men with biomarker and blood lead data, a total of 405 men also had bone lead measurements (Figure 1). After exclusion for baseline presence of heart disease, diabetes, or hs-CRP > 10 mg/L, 230 men remained with a total of 371 observations available for bone lead analysis. For all analyses, we estimated associations with the bone lead measurement taken closest in time to the biomarker measurement being analyzed. All participants provided written informed consent, and the study protocol was approved by the human research committees of Brigham and Women’s Hospital, and the VA Boston Healthcare System.

*Biomarker measurement.* Serum samples were drawn after overnight fasting. Serum cholesterol was assayed over the course of the study period using standard enzymatic methods and reagents (SCALVO Diagnostics, Wayne, NJ). The HDL fraction was measured in the supernatant after precipitation of the LDL and very low-density lipoprotein fractions with dextran sulfate and magnesium, using the Abbott Biochromatic Analyzer 100 (Abbott Laboratories, South Pasadena, CA). Triglyceride levels were measured using the Dupont ACA discrete clinical analyzer (Dupont Company, Biomedical Products Department, Wilmington, DE). LDL was estimated using the Friedewald equation (Friedewald et al. 1972).

The inflammatory markers for all time points were analyzed on archived samples stored at –80°C. Samples from each participant were analyzed in duplicate and in a single batch to avoid between-batch analytical variation. The performance of the assays was monitored with standard quality control procedures, including the analysis of quality control samples in each batch. Serum hs-CRP was measured using an immunoturbidimetric assay on the Hitachi 917 analyzer (Roche Diagnostics, Indianapolis, IN) with reagents and calibrators from DenkaSeiken (Niigata, Japan). Soluble ICAM-1 was measured using a quantitative sandwich enzyme-linked immunosorbent assay (R&D Systems, Minneapolis, MN). Sensitivity of the assay for ICAM-1 was 0.35 ng/mL, and the day-to-day variabilities of the assay at concentrations of 64.2, 117, 290, and 453 ng/mL were 10.1%, 7.4%, 6.0%, and 6.1%, respectively. IL-6 and soluble TNF-R2 were assayed with the Milliplex Map Human Cytokine/Chemokine Kit (Millipore Corp., St. Charles, MO) and detected with a multiplex detection platform (Luminex® 100/200™ System; Luminex Corporation, Austin, TX). The intraassay coefficients of variation for IL-6 and TNF-R2 were 10.6% and 5.9%, respectively.

*Lead measurement.* Blood lead was analyzed over the course of the study period by graphite furnace atomic absorption with Zeeman background correction (ESA Laboratories, Chelmsford, MA). The instrument was calibrated after every 21 samples with National Institute of Standards and Technology (NIST; Gaithersburg, MD) standard reference material (SRM 955a, lead in blood). Ten percent of the samples were run in duplicate, at least 10% as controls, and 10% as blanks. Compared with reference samples from the Centers for Disease Control and Prevention (CDC; Atlanta, GA), the precision ranged from 8% for concentrations < 30 µg/dL to 1% for higher concentrations.

Bone lead was measured for 30 min each at the mid-tibia shaft and at the patella using a K-shell X-ray fluorescence instrument (ABIOMED Inc., Danvers, MA). The technical specifications and validity of this instrument are described in detail elsewhere (Burger et al. 1990; Hu et al. 1990, 1994). The turnover is slower in cortical bone than in trabecular bone; thus, the half-life of lead in cortical bone versus trabecular bone is approximately 20 versus 8 years (Hu et al. 1991). The shaft of the tibia is mostly cortical bone, and the patella bone is mostly trabecular bone. Whereas bone lead levels represent cumulative exposure, blood lead levels represent acute exposure.

*Statistical analysis.* We evaluated mean baseline (time of the first biomarker measure) characteristics and biomarker levels according to blood lead quartiles using generalized linear models. Cut points for blood lead quartiles were based on the distribution in the largest sample. Given the variability in sample sizes for the biomarkers, we also performed sensitivity analyses for each biomarker by including only those participants who had all biomarkers measured (most participants excluded from the sensitivity analysis did not have a measure of TNF-R2). Additionally, we determined unadjusted Spearman rank correlations between baseline lead and biomarker levels. Then for all observations, we examined the relationship in age-adjusted models and multivariable models using generalized linear mixed models (with logit link) for dichotomized biomarkers (as described below) and with mixed-effects models for continuous biomarkers. We used the likelihood ratio test to select the compound symmetry covariance matrix with random intercept modeled in the following forms:

Dichotomized outcome: η*_ij_* = β_0_ + *u_i_* + β_1_age*_ij_* + β_2_lead*_ij_* + . . . + β*_p_X_pij_*, [1]

Continuous outcome: *Y_ij_* = β_0_ + *u_i_* + β_1_age*_ij_* + β_2_lead*_ij_* + . . . + β*_p_X_pij_* + ε*_ij_*, [2]

where η*_ij_* = log(*p_ij_* /1 – *p_ij_*) and represents the proportion of high biomarker outcomes (e.g., probability of being in the high-risk category for cholesterol) in subject *i* on visit *j*; *Y_ij_* is the biomarker level [e.g., log(total cholesterol level) in subject *i* on visit *j*]; β_0_ is the overall intercept; *u_i_* is the separate random intercept representing the subject-specific correlation among measurements; and β_1_ through β*_p_* represent the fixed effects for up to *p* covariates.

As a sensitivity analysis to adjust for the possibility that healthier men were more likely to return for subsequent exams, we weighted the findings by including an inverse probability of multiple exams (i.e., a revisit propensity score). The revisit propensity score was calculated from a logistic regression of the probability of not having two or more study visits, given all relevant factors including age, measurement of a particular marker (lipid profile or inflammatory marker), and total cholesterol level as well as diabetes, hypertension, and smoking status.

To assess the potential modifying effects of change in age from baseline age for each biomarker over time, we ran models that included a cross-product term for the interaction between change in age (time) and the preceding blood lead measure [lag blood lead (lag_lead_)], along with the main effects. For the first visit, we used the blood lead level prior to the inflammatory marker measurements. We also included the concurrent blood lead measure in the model because it is most likely more relevant to the acute biomarker levels, using the following form (e.g., for continuous outcome):

*Y_ij_* = *b*_0_ + *u_i_*+ *b*_1_*X*_age,_*_ij_* + β_2_lead*_ij_* + β_3_lag_lead,_*_ij_* + β_4_time*_ij_* + β_5_(lag_lead,_*_ij_* × time*_ij_*) + . . . + β*_p_X_pij_* + ε*_ij_*. [3]

Separate analyses considered biomarkers as continuous (log transformed to address issue of normality) or as categorical measures. We categorized the biomarkers to tie results to clinically relevant thresholds where possible. The lipid measurements were dichotomized based on guidelines for recommended levels from the Third Report of the U.S. National Cholesterol Education Program Adult Treatment Panel (ATP III): total cholesterol ≥ 200 mg/dL, LDL ≥ 130 mg/dL, HDL < 40 mg/dL, and triglycerides ≥ 200 mg/dL (Expert Panel 2001). hs-CRP was divided based on the American Heart Association (AHA)/CDC Scientific Statement (Pearson et al. 2003) at ≥ 3.1 mg/L (i.e., high risk for CVD). ICAM-1, TNF-R2, and IL-6, which do not have clinical cutoffs, were divided at their medians (281 ng/mL, 5.52 ng/mL, and 28.0 pg/mL, respectively). For these analyses, we reported results as odds ratios (ORs) for a 50% increase in lead exposure [i.e., exp(β_log[lead]_ × ln[1.5])]. The results for continuous outcomes were reported as the ratio of the geometric means (GMs) for a 50% increase in lead exposure [i.e., 1.5^(β_log[lead]_)]. Lead measures were modeled as log-transformed variables to address issues of normality. For analyses assessing the potential modifying effects of baseline age and change in age (interaction analyses), lead first was modeled continuously and then dichotomized at the median for ease of interpretation. All analyses were performed using SAS software, version 9.2 (SAS Institute Inc., Cary, NC).

Covariates of interest included baseline age (years), change in age from baseline over the follow-up period, and baseline measures of body mass index (BMI; kilograms per meter squared], education attainment level (< 12th grade, 12th grade, > 12th grade to < 4 years college/technical school, or ≥ 4 years of college), hypertension [physician diagnosis of hypertension with treatment or systolic blood pressure > 140 mmHg or diastolic blood pressure > 90 mmHg during examination (Chobanian et al. 2003)], statin use (yes or no), smoking status (current, former, or never), pack-years of smoking, and alcohol consumption (< 2 vs. ≥ 2 drinks/day). Physical activity was measured as metabolic equivalent of task (MET). A *p*-value of < 0.05 was considered significant, and a *p*-value of < 0.10 was considered marginally significant.

## Results

Characteristics of the study participants by quartile of blood lead level are presented in Table 1. At baseline, men with higher blood lead levels were more likely to be current smokers, consume ≥ 2 drinks a day, and be less physically active. When the sample was limited to participants with TNF-R2 measurements, sample differences across covariates were in the same direction but attenuated with regard to significance for alcohol consumption and physical activity (data not shown). Table 2 shows the baseline CVD biomarker concentrations by quartile of blood lead level. Those with higher blood lead levels were more likely to have significantly higher total cholesterol, HDL, and ICAM-1 levels. As with smoking and less physical activity, the results were the same when the sample was limited to participants with TNF-R2 measurements. The mean ± SD concentration was 4.01 ± 2.30 µg/dL for blood lead, 26.2 ± 18.8 mg/g for patella lead, and 19.8 ± 13.2 mg/g for tibia lead.

**Table 1:** Distribution of baseline characteristics across quartiles of blood lead (mean ± SD, or %).

Baseline blood lead (µg/dL)						
Variables	≤ 2.0	3.0	4.0	≥ 6.0	*p*-Value*a*
*n*		111		95		95		125		
Age (years)		71.0 ± 6.29		70.7 ± 7.10		72.3 ± 7.13		72.6 ± 6.52		0.10
BMI (kg/m^2^)		27.5 ± 3.64		27.3 ± 2.98		28.1 ± 3.88		27.8 ± 3.65		0.41
> High school education		96.4		98.9		96.8		91.9		0.07
Pack-years smoking		13.8 ± 18.8		21.1 ± 33.0		21.4 ± 24.4		23.7 ± 28.1		0.04
Current smoker		1.82		5.26		3.16		10.0		0.04
Two or more drinks		10.0		24.2		22.1		25.0		0.02
Physical activity (MET-hr/week)		14.5 ± 17.8		20.3 ± 27.5		16.0 ± 17.2		11.8 ± 15.7		0.02
Hypertensive		57.3		56.8		59.0		71.0		0.08
Statin use		24.5		13.7		22.1		16.1		0.16
**a**Determined using generalized linear equation.										

**Table 2:** Distribution of baseline levels of lipid profile and biomarkers of inflammation across quartiles of baseline blood lead (mean ± SD).*^a^*

Baseline blood lead (µg/dL)						
Variable	≤ 2.0	3.0	4.0	≥ 6.0	*p*-Value*b*
*n*		111*c*		95*d*		95*e*		124*f*		
Lipid profile (mg/dL)										
Total cholesterol		201 ± 33.9		208 ± 34.8		216 ± 36.9		217 ± 36.2		0.002
LDL		127 ± 29.1		130 ± 30.6		133 ± 28.9		136 ± 33.8		0.12
HDL		47.7 ± 12.4		52.6 ± 13.3		53.8 ± 14.4		52.7 ± 13.7		0.004
Triglycerides		130 ± 61.4		131 ± 81.7		141 ± 109		140 ± 82.3		0.71
Inflammation										
hs-CRP (mg/L)		1.86 ± 1.72		1.89 ± 1.90		2.01 ± 1.92		2.42 ± 2.09		0.11
ICAM-1 (ng/mL)		271 ± 54.4		268 ± 64.4		281 ± 70.4		300 ± 90.2		0.03
IL-6 (pg/mL)		183 ± 319		276 ± 569		123 ± 176		154 ± 326		0.14
TNF-R2 (ng/mL)		5.51 ± 2.14		5.32 ± 1.84		5.55 ± 1.69		6.09 ± 2.22		0.14
**a**Quartile cut points are the same for all sample sizes. **b**Determined using generalized linear equation. **c**For TNF-R2 and IL-6, *n* = 66. **d**For TNF-R2 and IL-6, *n* = 56. **e**For TNF-R2 and IL-6, *n* = 58. **f**For LDL, HDL, and triglycerides, *n* = 123; for TNF-R2 and IL-6, *n* = 73.										

Tibia lead levels did not correlate with any of the CVD biomarkers, and patella lead levels were significantly correlated only with HDL (Table 3). In contrast, blood lead was significantly and positively correlated with total cholesterol, LDL, HDL, hs-CRP, ICAM-1, and TNF-R2. Therefore, we focused the remainder of our analysis on the relationship of blood lead levels with CVD biomarkers.

**Table 3:** Spearman rank correlations (*r*_S_) of baseline lead concentrations and baseline levels of lipid profile and biomarkers of inflammation.

Biomarker	Blood lead	Patella lead	Tibia lead
Lipid profile						
Total cholesterol						
*r*_S_		0.18		0.03		0.06
*p*-Value		< 0.001		0.70		0.38
*n*		425		230		230
LDL*a*						
*r*_S_		0.12		–0.02		0.04
*p*-Value		0.01		0.80		0.51
*n*		424		229		229
HDL						
*r*_S_		0.15		0.18		0.05
*p*-Value		0.002		0.01		0.41
*n*		424		229		229
Triglycerides						
*r*_S_		0.03		–0.07		0.04
*p*-Value		0.51		0.32		0.59
*n*		424		229		229
Inflammation						
hs-CRP						
*r*_S_		0.13		0.05		0.05
*p*-Value		0.01		0.45		0.41
*n*		426		230		230
ICAM-1						
*r*_S_		0.13		0.02		–0.06
*p*-Value		0.01		0.73		0.33
*n*		425		229		229
IL-6						
*r*_S_		–0.04		0.06		–0.05
*p*-Value		0.58		0.51		0.55
*n*		253		139		139
TNF-R2						
*r*_S_		0.13		0.13		0.13
*p*-Value		0.03		0.14		0.12
*n*		253		139		139
**a**Calculated from total cholesterol, HDL, and triglycerides for those with triglycerides < 400 mg/dL.						

In age-adjusted models using the repeated measures, higher blood lead was significantly associated with the high-risk categories for total cholesterol, hs-CRP, and TNF-R2 and the low-risk category for HDL (Table 4). After adjusting for the full set of covariates, the associations with total cholesterol and HDL were no longer significant, and the association with hs-CRP was attenuated, becoming marginally associated (*p* = 0.09). Associations with TNF-R2 remained significant and similar in magnitude. The relationship was maintained after further including the revisit propensity score. A 50% increase in blood lead level was associated with a 26% increased likelihood of having high TNF-R2 levels [OR = 1.26; 95% confidence interval (CI): 1.09, 1.45].

**Table 4:** Multivariate analyses with age*^a^*- and multivariable*^b^*-adjusted outcomes associated with a 50% increase in blood lead for high-risk biomarkers*^c^* [OR (95% CI)] and log-transformed continuous biomarkers [ratios of GMs (95% CI)].

Biomarker	OR (95% CI)	*p*-Value	Ratio of GMs (95% CI)	*p*-Value
Lipid profile								
Total cholesterol (total, *n* = 620; high, *n* = 335)								
Age adjusted		1.19 (1.04, 1.36)		0.01		1.02 (1.01, 1.03)		< 0.001
Multivariable adjusted		1.13 (0.98, 1.30)		0.09		1.01 (1.00, 1.02)		0.01
Further weighted for inverse probability of revisits		1.12 (0.97, 1.28)		0.11		1.01 (1.00, 1.02)		0.01
LDL*d* (total, *n* = 614; high, *n* = 277)								
Age adjusted		1.10 (0.97, 1.25)		0.14		1.02 (1.01, 1.04)		0.002
Multivariable adjusted		1.03 (0.90, 1.18)		0.61		1.01 (1.00, 1.03)		0.06
Further weighted for inverse probability of revisits		1.03 (0.91, 1.18)		0.59		1.01 (1.00, 1.03)		0.06
HDL (total, *n* = 619; high, *n* = 124)								
Age adjusted		0.83 (0.76, 0.98)		0.02		1.02 (1.01, 1.04)		< 0.001
Multivariable adjusted		0.85 (0.72, 1.01)		0.11		1.02 (1.01, 1.03		0.002
Further weighted for inverse probability of revisits		0.88 (0.74, 1.04)		0.13		1.02 (1.01, 1.03)		0.002
Triglycerides (total, *n* = 619; high, *n* = 77)								
Age adjusted		1.03 (0.86, 1.22)		0.72		0.99 (0.97, 1.02)		0.63
Multivariable adjusted		0.99 (0.82, 1.21)		0.78		0.99 (0.96, 1.02)		0.47
Further weighted for inverse probability of revisits		0.97 (0.81, 1.18)		0.91		0.99 (0.96, 1.02)		0.47
Inflammation								
hs-CRP (total, *n* = 621; high, *n* = 127)								
Age adjusted		1.23 (1.07, 1.44)		0.01		1.06 (1.01, 1.12)		0.03
Multivariable adjusted		1.14 (0.97, 1.33)		0.10		1.03 (0.98, 1.09)		0.23
Further weighted for inverse probability of revisits		1.14 (0.98, 1.34)		0.10		1.03 (0.98, 1.09)		0.20
ICAM-1 (total, *n* = 620; high, *n* = 283)								
Age adjusted		1.04 (0.84, 1.18)		0.53		1.01 (1.00, 1.03)		0.05
Multivariable adjusted		1.02 (0.89, 1.16)		0.88		1.01 (1.00, 1.02)		0.14
Further weighted for inverse probability of revisits		1.00 (0.89, 1.13)		0.96		1.01 (1.00, 1.03)		0.12
IL-6 (total, *n* = 430; high, *n* = 207)								
Age adjusted		0.97 (0.84, 1.13)		0.73		1.02 (0.89, 1.17)		0.76
Multivariable adjusted		0.98 (0.81, 1.15)		0.91		1.05 (0.91, 1.20)		0.54
Further weighted for inverse probability of revisits		0.99 (0.86, 1.14)		0.95		1.04 (0.90, 1.20)		0.60
TNF-R2 (total, *n* = 430; high, *n* = 203)								
Age adjusted		1.24 (1.07, 1.44)		0.004		1.05 (1.02, 1.08)		< 0.001
Multivariable adjusted		1.28 (1.10, 1.50)		0.002		1.05 (1.03, 1.08)		< 0.001
Further weighted for inverse probability of revisits		1.26 (1.09, 1.45)		0.002		1.05 (1.02, 1.08)		< 0.001
**a**Adjusted for age at baseline and difference in age between baseline and time outcome was measured. **b**Additionally adjusted for education, BMI, alcohol intake, pack-years of smoking, smoking status, hypertension status, and statin use. **c**Based on ATP III guidelines for lipid profile [total cholesterol ≥ 200; LDL ≥ 130 mg/dL; HDL < 40 mg/dL (low levels of good cholesterol); triglycerides ≥ 200 mg/dL (Expert Panel 2001)] and on the AHA/CDC recommendation for C-reactive protein (hs-CRP) ≥ 3.1 mg/L. (Pearson et al. 2003). For biomarkers without recommendation based on the median: ICAM-1 > 281 ng/mL, TNF-R2 > 5.52 ng/mL, and IL-6 > 28.0 pg/mL. **d**Calculated from total cholesterol, HDL, and triglycerides for those with triglycerides < 400 mg/dL.								

For continuous log-transformed outcomes in age-adjusted models, higher blood lead was significantly associated with higher total cholesterol, LDL, and higher HDL, as well as higher hs-CRP and TNF-R2 (Table 4). In multivariable and weighted multivariable analyses, the association was no longer maintained for hs-CRP. The associations remained significant for total cholesterol, HDL, and TNF-R2. For example, a 50% increase in blood lead level was associated with a 5% increase in the GM of TNF-R2 (ratio of GMs = 1.05; 95% CI: 1.02, 1.08).

We also evaluated whether lag_lead_ modified the odds of high TNF-R2 (categorical) over time and the rate of change of TNF-R2 (continuous) over time, controlling for concurrent blood lead (Table 5). We did not observe a significant estimated effect of lag_lead_ in the odds of high TNF-R2 across time, but there was an association with the rate of change of TNF-R2 over time. Those with higher than median blood lead at the preceding visit had a discernibly greater increase in TNF-R2 levels over time than those with low blood lead. Specifically, compared with those with low blood lead, those with high blood lead had an additional 0.03 ng/mL increase in log TNF-R2 (i.e., additional 1% increase in GM of TNF-R2) across time (*p*-interaction = 0.01).

**Table 5:** Longitudinal analysis of age*^a^*- and multivariable*^b^*-adjusted OR and ratio of GMs of difference in the relationship with a 50% increase in lag_lead_ and high and log-transformed TNF-R2, respectively, with change in time after baseline.

Adjustment	OR (95% CI)	*p*-Value	Ratio of GMs (95% CI)	*p*-Value
Age adjusted		1.02 (0.96, 1.08)		0.45		1.01 (1.00, 1.02)		0.01
Multivariate adjusted		1.03 (0.98, 1.08)		0.28		1.01 (1.00, 1.02)		0.01
Further weighted for inverse probability of revisits		1.02 (0.96, 1.09)		0.44		1.01 (1.00, 1.02)		0.01
**a**Adjusted for age at baseline, difference in age between baseline and time outcome, concurrent blood lead, lag_lead_, and difference in age × high lag_lead_. **b**Additionally adjusted for education, BMI, alcohol intake, pack-years of smoking, smoking status, hypertension status, and statin use.								

## Discussion

Longitudinal analysis demonstrated a positive association of blood lead levels with TNF-R2. The relationship with TNF-R2 and blood lead significantly increased with change in age from baseline; that is, higher blood lead was associated with a steeper subsequent rate of increase in TNF-R2 over time. The associations were unchanged even after adjusting for an array of medical conditions and biological and behavioral factors, as well as accounting for a higher propensity of those in better health to have repeated measures. Also, blood lead was positively associated with the lipid markers total cholesterol, LDL, and HDL; however, effect estimates were attenuated in multivariable models, particularly for outcomes categorized using clinical cut points. In age-adjusted models, blood lead was significantly associated with hs-CRP, but the association was not significant after further adjustment.

Lead exposure has been associated with adverse effects on cardiovascular health. However, as far as we know, no other study has comprehensively evaluated the relationship between lead and potential mediating biological processes involved in CVD or how these processes play out over time (i.e., with age). Potential mediating processes may be assessed with biomarkers that indicate lipid profile and levels of inflammation or endothelial dysfunction (Pearson et al. 2003; Selhub 2006). Approximately 95% of lead in adults is stored in bone. It has been hypothesized that bone lead is continuously released into blood, with the rate of mobilization differing by age and bone type, and that the mobilized lead contributes to adverse health effects (Hu et al. 1998). We would expect that these acute biomarkers would be most closely related to the acute marker of lead (blood lead), which is consistent with our findings.

In the present study, our strongest finding was that initial blood lead levels were associated with more rapid rise in inflammation as measured by TNF-R2 over time. In the inflammatory cascade model proposed by Pearson et al. (2003), primary proinflammatory cytokines such as TNF-α can have a direct impact on circulation by increasing the products of hepatic stimulation such as CRP or by influencing the levels of adhesion molecules. TNF-α functions as a mediator of inflammatory reactions and plays a part in the immunopathogenesis of hypertensive disease (Dorffel et al. 1999). Dysregulation of the expression of TNF-α is associated with vascular aging and leads to vascular oxidative stress and remodeling, thrombosis, cell infiltration, apoptosis, and vascular inflammation (Zhang et al. 2009). Our results agree with those of Valentino et al. (2007), who found a positive relationship between TNF-α and blood lead in low-lead–exposed workers versus nonexposed workers. Lead appears to stimulate TNF-α production by modifying the expression of TNF-R2 through its interaction with cysteine-rich membrane proteins. The observed interaction of high baseline blood lead with changes in TNF-R2 seems to indicate a modifying effect of blood lead on the normal age-related increase in the levels of proinflammatory markers, similar to the effects of environmental stimulation [e.g., lipopolysaccharide (LPS)] on TNF-α. In an animal study, Guo et al. (1996) found that lead potentiated the lethality of LPS by increasing expression of both TNF-α and TNF-R2. This age-related increase in response to environmental stimulus is thought to be due to alterations in Toll-like receptor function, a protective mechanism involved in limiting inflammation (Larbi et al. 2008; May et al. 2009; van Duin and Shaw 2007).

We found some evidence of an association of blood lead with CRP and ICAM-1 in unadjusted models, suggesting that effects on these inflammatory and endothelial functions are possible but might be an artifact of effects on other related processes. A small occupational study of 87 lead-exposed workers and 61 nonexposed workers matched on age and sex also found a relationship between blood lead and CRP in lead-exposed workers in bivariate models (Khan et al. 2008). However, another population-based study did not find a dose-dependent relationship between blood lead level and CRP after adjusting for a broad array of potential confounders (Songdej et al. 2010), similar to our findings. Moreover, similar to reports by Valentino et al. (2007), we did not see any evidence of an effect of lead on IL-6 levels.

We also observed an association between blood lead and total cholesterol based on an age-adjusted model, but the association was attenuated after controlling for additional covariates. In analyses of continuous (log-transformed) HDL (rather than HDL categorized by clinical guidelines), there was also a significant increase in HDL with higher blood lead levels. Similar findings have been reported for lead-exposed patients by Cocco et al. (1991), but they also saw decreases in total cholesterol and LDL with lead exposure. However, another study of occupational lead exposure found a positive correlation between blood lead and total cholesterol and LDL, but not between blood lead and HDL (Ademuyiwa et al. 2005). In one study with rats, low doses of lead were associated with a decrease in total cholesterol and HDL and an increase in triglyceride levels (Skoczynska et al. 1993); however, in contrast to humans, rats transport the major proportion of plasma cholesterol by HDL (Hassan 1987; Skoczynska et al. 1993). Emerging research has found that although HDL is generally considered protective against CVD, under certain situations it may not be atheroprotective (Feng and Li 2009). Additionally, it is still not clear whether lead induces lipid abnormalities by altering lipid metabolism (e.g., increasing serum cholesterol levels) or by stimulating lipid peroxidation (Skoczynska et al. 1993).

This study has several limitations. The sample size was relatively small. Although we had approximately 636 observations, the available sample size varied by biomarker. In addition, we may have inadequately controlled for or missed potential confounders. Moreover, in this report, we focused on the acute measure of lead exposure: blood lead. Although we chose this focus because most of the univariable relationships were observed with blood lead, the comparison with a marker of acute lead exposure (i.e., blood lead) may be appropriate when evaluating relationships with serum biomarkers. We also used lag_lead_ measured at the previous visit (usually 3–4 years before the biomarker data) to look at change over time in biomarker levels. The analysis was modeled in this way because we wanted to be sure the exposure preceded the change in biomarker concentrations. We note that similar results were obtained whether we used concurrent or lag_lead_ in the interaction term (data not shown). Finally, this study was of males only, and for some of these biomarkers, the literature suggests that their relationships with CVD may vary between men and women (e.g., Jenny et al. 2006; Lim and Cassano 2002). However, this study was unique in that it allowed us to evaluate acute and cumulative lead exposure on multiple cardiovascular risk factors and risk markers. To our knowledge, this is also the first study to examine changes in associations between lead and biomarkers over time. Although the comprehensive list of biomarkers allowed us to investigate pathways of action at different stages along the ischemic cascade, it may raise concerns about multiple comparisons. Given mixed findings from prior studies, our results involving the lipid-related variables may warrant further investigation. However, for TNF-R2, the magnitude of effect and consistency of findings cross-sectionally and longitudinally support the possibility that the association between lead and TNF-R2 is causal.

Our primary findings are that blood lead levels are associated with TNF-R2 levels. In addition, higher blood lead exposure at the preceding visit is associated with an increase in the change in TNF-R2 with age. This indicates that lead may play a role in increasing inflammation and may potentiate age-related increases in inflammation.
